# The complete chloroplast genome of *Plagiorhegma dubia* Maxim., a traditional Chinese medicinal herb

**DOI:** 10.1080/23802359.2018.1424586

**Published:** 2018-01-10

**Authors:** Caihong Wang, Ying Wang, Qiong Liang, Tae-Jin Yang, Zhilin Li, Yanjun Zhang

**Affiliations:** a College of Horticulture and Landscape, Yunnan Agriculture University, Kunming, People’s Republic of China;; b Key Laboratory of Plant Germplasm Enhancement and Specialty Agriculture, Wuhan Botanical Garden, Chinese Academy of Sciences, Wuhan, People’s Republic of China;; c Key Laboratory of Plant Resources Conservation and Sustainable Utilization & Guangdong Provincial Key Laboratory of Applied Botany, South China Botanical Garden, Chinese Academy of Sciences, Guangzhou, People’s Republic of China;; d Department of Plant Science, Plant Genomics and Breeding Institute, Research Institute of Agriculture and Life Sciences, College of Agriculture and Life Sciences, Seoul National University, Seoul, Republic of Korea

**Keywords:** Chloroplast, *Plagiorhegma dubia*, genome sequence, medicinal herb

## Abstract

*Plagiorhegma dubia* Maxim. is a traditional Chinese medicinal herb from *Plagiorhegma*, Berberidaceae, which is distributed in the northeast of China, Korea, Russia. The complete chloroplast genome is 152,468 bp in length, with large single copy (LSC 82,257 bp) and small single copy (SSC 16,599 bp) regions separated by a pair of inverted repeats (IR 26 805 bp). The genome has a total of 113 genes including 79 protein-coding genes, 30 tRNA genes, and 4 rRNA genes. Phylogenetic analysis shows that *P. dubia* is closely related with *Sinopodophyllum hexandrum* and *Epimedium* species. The results are of great implication for the development and utilization of *P. dubia* and the phylogenetic researches on Berberidaceae.

*Plagiorhegma dubia* Maxim is a perennial herbaceous plant and the only species of the genus *Plagiorhegma* of Berberidaceae, Ranales, which is distributed in Northeast China, Korea, Russia (Ying et al. 2011). As the traditional Chinese medicinal material, the rhizome of *P. dubia* contains berberine, and has antipyretic, antidotal, stomachic and antidiarrhoeal clinical effects (Arens et al. 1985; Jeong and Sivanesan 2016). Furthermore, *P. dubia* has potential as a new ornamental garden plant which has attractive heart-shaped leaves and light purple flowers. The species has potential as a new ornamental garden plant (Rhie et al. 2014).

The total genomic DNA of *P. dubia* was extracted from its fresh leaves which were collected in Tonghua of Jinlin Province, China (N 41°43′48″ E 125°59′24″). An Illumina paired-end (PE) genomic library was prepared and sequenced. High quality reads were obtained from the total pair-end (PE) raw reads and assembled by the CLC-quality genome assembler (ver 4.06bata) (Kim et al. 2015). All of the contig sequences were aligned and ordered to the reference cp genome of *Nandina domestica* (NC_DQ923117) using MUMmer (Kurtz et al. 2004). Sequence gaps were filled by Gapcloser included in the SOAP package vl.12 (Li et al. 2010). Four junctions between LSC/IRs and SSC/IRs were validated with PCR-based conventional Sanger sequencing.

The genes in the chloroplast genome were predicted by the Dul Organellar GenoMe Annotator (DOGMA; Wyman et al. 2004). The BLAST tools and ORF finder at NCBI website (http://www.ncbi.nlm.nih.gov/) were also used in the gene annotations. The tRNA genes were verified with tRNAscan-SE (Lowe and Eddy 1997). The circular cp genome maps were drawn by the Organellar Genome DRAW tool (OGDRAW; Lohse et al. 2007) with subsequent manual editing.

The complete chloroplast genome of *P. dubia* was submitted to NCBI, and the accession number of nucleotide sequence is MG397139. The nucleotide sequence was 152,468 bp, and was assembled into a single circular which presented a typical quadripartite structure including one large single-copy region (82,257 bp), one small single copy (16,599 bp), and a pair of inverted repeat regions (IRa and IRb) of 26805 bp. The GC content of the chloroplast genomes was 38.15%. The 113 unique genes in the sequence were composed of 79 protein-coding genes, 30 tRNA genes and four rRNA genes.

The phylogenetic analyses sampled *P. dubia* and other nine Berberdaceae species. These nine species had been sequenced on chloroplast genome, including four *Epimedium* species (Lee et al. 2015; Sun et al. 2016; Zhang et al. 2016), *Sinopodophyllum hexandrum* (Meng et al. 2017), two *Berberis* species (NC_KM057374, NC_KM057375), *Nandina domestica* (Moore et al. 2006), *Gymnosepermium microrrhynchum* (NC_KM057373). ML phylogenetic tree was constructed based on the entire chloroplast protein-coding sequences of these 11 species using MEGA7.0 (Kumar et al. 2016). The results showed that the ten Berberidaceae species grouped into a monophyletic branch (Figure 1). In accordance with previous phylogenetic studies on Berberidaceae (Kim and Jansen 1994; Wang et al. 2007; Zhang et al. 2012), inter-genera relationships of the family were closely related with chromosome base number. *N. domestica* with *x* = 10 and *G. microrrhynchum* with *x* = 8 clustered into a clade. *Berberis amurensis* and *B. korena* are with *x* = 7 and formed a branch. *Epimedium koreanum*, *E. sagittatum*, *E. lishichenii*, *E. pseudowushanense*, *S. hexandrum, P. dubia* are with *x* = 6 and had most closest relationships. The results are of great implication for the Phylogenetic researches on Berberidaceae.

**Figure 1.**
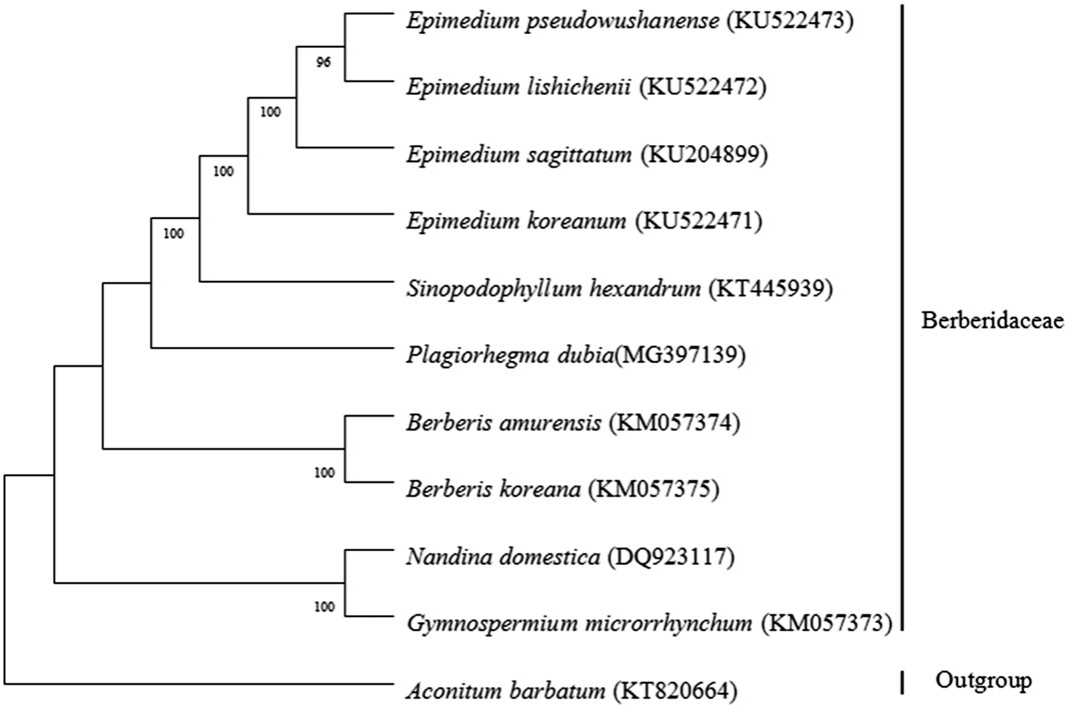
Using MEGA7.0, ML phylogenetic tree was constructed based on the entire chloroplast protein-coding sequences of ten Berberdaceae species including *P. dubia* and *Aconitum barbatum* of Ranunculaceae as an outgroup.

## References

[CIT0001] Arens H, Fischer H, Leyck S, Römer A, Ulbrich B. 1985 Antiinflammatory compounds from *Plagiorhegma dubium* cell culture. Planta Medica. 51:52–56. 4011754

[CIT0002] Jeong BR, Sivanesan I. 2016 Micropropagation, berberine content and antitumor activity of *Jeffersonia dubia* (Maxim.) Benth et Hook. Plant Cell Tissue Organ Culture. 124:453–458.

[CIT0003] Kim K, Lee SC, Lee J, Lee HO, Joh HJ, Kim NH, Park HS, Yang TJ. 2015 Comprehensive survey of genetic diversity in chloroplast genomes and 45s nrDNAs within *Panax ginseng* species. PLoS One. 10:e0117159. 26061692 10.1371/journal.pone.0117159 PMC4465672

[CIT0004] Kim YD, Jansen RK. 1994 Characterization and phylogenetic distribution of a chloroplast DNA rearrangement in the Berberidaceae. Plant System Evol. 193:107–114.

[CIT0005] Kumar S, Stecher G, Tamura K. 2016 Mega 7: molecular evolutionary genetics analysis version 7.0 for bigger datasets. Mol Biol Evol. 33:1870–1874. 27004904 10.1093/molbev/msw054 PMC8210823

[CIT0006] Kurtz S, Phillippy A, Delcher AL, Smoot M, Shumway M, Antonescu C, Salzberg SL. 2004 Versatile and open software for comparing large genomes. Genome Biol. 5:R12. 14759262 10.1186/gb-2004-5-2-r12 PMC395750

[CIT0007] Lee JH, Kim K, Kim NR, Lee SC, Yang TJ, Kim YD. 2015 The complete chloroplast genome of a medicinal plant *Epimedium koreanum* Nakai (Berberidaceae). Mitochondrial DNA Part A. 27:4342–4343. 10.3109/19401736.2015.1089492 26462716

[CIT0008] Li RQ, Zhu HM, Ruan J, Qian WB, Fang XD, Shi ZB, Li Y, Li S, Shan G, Kristiansen K, et al 2010 De novo assembly of human genomes with massively parallel short read sequencing. Genome Research. 20:265–272. 20019144 10.1101/gr.097261.109 PMC2813482

[CIT0009] Lohse M, Drechsel O, Bock R. 2007 Organellar Genome DRAW (OGDRAW): a tool for the easy generation of high-quality custom graphical maps of plastid and mitochondrial genomes. Current Genetics. 52:267–274. 17957369 10.1007/s00294-007-0161-y

[CIT0010] Lowe TM, Eddy SR. 1997 tRNAscan-SE: a program for improved detection of transfer RNA genes in genomic sequence. Nucleic Acids Res. 25:955–964. 9023104 10.1093/nar/25.5.955 PMC146525

[CIT0011] Meng LH, Liu RJ, Chen JB, Ding CX. 2017 The complete chloroplast genome of *Sinopodophyllum hexandrum* Ying (Berberidaceae). Mitochondrial DNA Part A. 28:342–343. 10.3109/19401736.2015.1122773 26713941

[CIT0012] Moore MJ, Dhingra A, Soltis PS, Shaw R, Farmerie WG, Folta KM, Soltis DE. 2006 Rapid and accurate pyrosequencing of angiosperm plastid genomes. BMC Plant Biol. 6:17. 16934154 10.1186/1471-2229-6-17 PMC1564139

[CIT0013] Rhie YH, Lee SY, Jung HH, Kim KS. 2014 Light intensity influences photosynthesis and crop characteristics of *Jeffersonia dubia*. Korean J Horticult Sci Technol. 32:584–589.

[CIT0020] Sun YX, Moore MJ, Zhang S, Soltis PS, Soltis DE, Zhao T, Meng A, Li X, Li J, Wang H. 2016 Phylogenomic and structural analyses of 18 complete plastomes across nearly all families of early-diverging eudicots, including an angiosperm-wide analysis of ir gene content evolution. Molecular Phylogenetics & Evolution. 96:93–101. 26724406 10.1016/j.ympev.2015.12.006

[CIT0014] Wang W, Chen ZD, Liu Y, Li RQ, Li JH. 2007 Phylogenetic and biogeographic diversification of Berberidaceae in the northern hemisphere. Systematic Bot. 32:731–742.

[CIT0015] Wyman SK, Jansen RK, Boor JL. 2004 Automatic annotation of organellar genomes with DOGMA. Bioinformatics. 20:3252–3255. 15180927 10.1093/bioinformatics/bth352

[CIT0016] Ying TS, Boufford DE, Brach AR. 2011 “Berberidaceae”, in Flora of China. Wu ZY, Raven PH, Hong DY, editors. Beijing: Science Press/St. Louis (MO): Missouri Botanical Garden Press; p. 714–801.

[CIT0017] Zhang MY, Lu L, Li DZ, Wang H. 2012 Evolution of the pollen in the family Berberidaceae. Plant Diversity Resour. 34:1–11.

[CIT0018] Zhang YJ, Du LW, Liu A, Chen JJ, Wu L, Hu WM, Zhang W, Kim K, Lee SC, Yang TJ. 2016 The complete chloroplast genome sequences of five *Epimedium* Species: lights into phylogenetic and taxonomic analyses. Frontiers Plant Sci. 7:306. 10.3389/fpls.2016.00306 PMC4791396 27014326

